# A New Multi-Sensor Stream Data Augmentation Method for Imbalanced Learning in Complex Manufacturing Process

**DOI:** 10.3390/s22114042

**Published:** 2022-05-26

**Authors:** Dongting Xu, Zhisheng Zhang, Jinfei Shi

**Affiliations:** 1 School of Mechanical Engineering, Southeast University, Nanjing 211189, China; xudongting@seu.edu.cn (D.X.); oldbc@seu.edu.cn (Z.Z.); 2 School of Mechanical Engineering, Nanjing Institute of Technology, Nanjing 211167, China

**Keywords:** imbalanced learning, data augmentation, multi-sensor stream data, multivariate time series, complex manufacturing process, supervised learning, failure detection

## Abstract

Multiple sensors are often mounted in a complex manufacturing process to detect failures. Due to the high reliability of modern manufacturing processes, failures only happen occasionally. Therefore, data collected in practical manufacturing processes are extremely imbalanced, which often brings about bias of supervised learning models. Data collected by the multiple sensors can be regarded as multivariate time series or multi-sensor stream data. The high dimension of multi-sensor stream data makes building models even more challenging. In this study, a new and easy-to-apply data augmentation approach, namely, imbalanced multi-sensor stream data augmentation (IMSDA), is proposed for imbalanced learning. IMSDA can generate high quality of failure data for all dimensions. The generated data can keep the similar temporal property of the original multivariate time series. Both raw data and generated data are used to train the failure detection models, but the models are tested by the same real dataset. The proposed method is applied to a real-world industry case. Results show that IMSDA can not only obtain good quality failure data to reduce the imbalance level but also significantly improve the performance of supervised failure detection models.

## 1. Introduction

Modern industry is changing from traditional manufacturing to artificial intelligence manufacturing along with the fast development of digital technologies. The production facilities and manufacturing processes which transform the requisite amounts of materials into finished products are substantial and technologically complex. Collecting data, training model, and deployment in production constitute a typical artificial intelligence (AI) lifecycle [1]. The principles of how to build an AI system are still developing. Most AI systems provide a fixed set of data, and engineers work on the iterations of the code. This allows us to find the model which can perform best by comparing algorithms running on the same dataset. However, there are a lot of tough challenges in real-world applications. If data are of poor quality, the decisions are likely to be unsound. Due to the high reliability of modern manufacturing processes, failures only happen occasionally. Therefore, data collected in practical manufacturing processes are usually imbalanced, which often brings about bias of supervised learning models. Imbalanced data significantly compromise the performance of most standard machine learning algorithms. The model often performs well in the lab but performs poorly in practice. However, there are not many efficient and effective techniques for building good quality of training datasets, which can have a big impact on the performance in practical applications. It is time to hold the code fixed and spur an equally large amount of innovation to improve the imbalanced data quality.

Big data analytics, such as machine learning or deep learning, have been a core technology to empower intelligent manufacturing systems for process control or fault detection [2]. It is hard to find the failure patterns of a production line, due to the lack of failure data. When presented with extreme imbalanced datasets, most standard learning algorithms fail to properly represent the characteristics of the data and resultantly provide unfavorable accuracies across the classes of the data. The imbalanced data will lead to low data utilization efficiency and even bring serious decision-making mistakes. Data augmentation is an important tool to generate high quality of synthetic data to balance the training data. Data augmentation is a technique that can increase the diversity of the training data and keep the affinity without collecting new data [3]. Data augmentation tools can be developed based on historical data, domain knowledge, or a combination of both. The techniques expand the training dataset with class-preserving transformations. Data augmentation has shown its effectiveness at improving data quality in many applications [4].

In advanced manufacturing processes, a lot of sensors are placed in different parts of the machines, and these sensors measure both raw materials and process variables. Most classical single-signal processing techniques which have many great achievements in failure detection lost their advantages in plantwide sensor data analysis. The high dimensionality of the multi-sensor stream data collected from the processes makes failure detection using supervised learning more challenging. Both unsupervised learning and supervised learning methods can be applied to the data, but the latter method needs enough failure data to avoid the bias caused by the imbalance of labeled failure data during the training progress. Data augmentation is an effective method to generate synthetic failure data for algorithm training. However, there are only a few studies on data augmentation which takes the plantwide variables into account for supervised imbalanced learning. Even the single-sensor stream data augmentation methods cannot be directly generalized to multi-sensor stream data [4].

Data augmentation techniques are widely used in image and audio signal processing, while the techniques can help to significantly improve the performance of classification [5,6]. A typical method, such as creating more synthetic labeled images to balance the original data, is a good idea for imbalanced learning. There are several data augmentation methods that work for imbalanced data, and some typical advanced data augmentation methods are selected for illustration. Sampling method is a typical solution to address the imbalanced learning issue. Undersampling removes the data from the original dataset, and it gives a simple method for adjusting the balance of the original dataset [7]. Oversampling augmentations create synthetic instances and add them to the training set [8]. SMOTE is a famous and sophisticated oversampling technique. It creates the synthetic minority class examples and makes the dataset balanced [9]. Some improved SMOTE-based methods are proposed later [10,11,12,13,14,15]. ADASYN (Adaptive Synthetic Sampling Approach for Imbalanced Learning) is designed for two-class classification. The key idea of it is using the density distribution of a parameter which is a measurement of different minority class examples according to their level of difficulty in learning to automatically decide the number of synthetic samples. The major difference compared to SMOTE is that ADASYN does not need to perform evaluation of hypothesis performance to update the distribution function [16]. However, most of these sampling approaches may change the temporal property of multivariate time series and may not be directly used on the multi-sensor stream data from complex manufacturing processes.

To solve the problems related with multivariate time series, the techniques concerning univariate time series are often consulted first. Time series data augmentation tools can be decomposition methods-based. A decomposition method such as STL (Seasonal-Trend Decomposition Procedure Based on Loess) or Robust-STL decomposes time series to trend signal and periodic signal [17,18]. Bootstrapping is proposed to apply on the STL decomposed residuals to generate augmented signals, which are then added back with trend and seasonality to assemble a new time series [19]. Time-domain and frequency-domain augmentation are applied on the decomposed residual, and they can help to increase the performance of anomaly detection significantly [20]. However, high dimensional multivariate time series data collected from multiple sensors which measure different machines and processes cannot always be decomposed into trend and seasonal signals in practice. This kind of decomposition method usually does not work for data fusion which integrates multiple data sources.

Recently, more and more data augmentation methods have been proposed [21,22]. Adding noise and interpolation are simple time series augmentation methods [23,24]. Some researchers have proposed several network generating methods for time series. For multivariate time series, the conditional generative adversarial network can be used to perform data-informed oversampling in order to balance the extreme imbalanced dataset [25]. The automatic augmentation policies such as the adaptive weighting scheme have achieved state-of-the-art performance on the time series datasets from the UCR archive [26]. However, the method is only useful for univariate time series; it has not shown its feasibility for deployment in multivariate time series in practice.

In recent years, augmentation strategies for heterogeneous multimodal inputs have been proposed to increase the diversity of the training samples, but the transformation functions proposed could not be directly used on the multi-sensor stream data for imbalanced learning [27]. A stream data augmentation method based on generative adversarial network has been proposed to improve the performance of sensor anomaly in industrial robots; however, the methods could not deal with the multi-sensor stream data from a production line in manufacturing [28]. These research works show that the task which combines multi-senor stream data augmentation and imbalanced learning is very difficult and challenging.

In practice, detecting or predicting failures in manufacturing processes is a classical problem in industries [29,30,31]. Nowadays, more and more control systems apply artificial intelligence models to analyze the data collected from the manufacturing processes [32,33]. Recently, deep learning methods, such as Autoencoder and LSTM (long-short-term-memory), have been used for fault detection and prediction in complex industrial processes [34,35]. However, they are unsupervised learning methods that do not use labeled failure data. Feature engineering is a commonly used method before building failure detection or quality control models in manufacturing processes [36,37,38]. Multi-sensor data fusion is also a widely used approach for process monitoring and anomaly detection [39]. In this study, a new imbalanced multi-sensor stream data augmentation method is proposed for failure detection using supervised learning in the manufacturing process.

There are some fundamental questions in imbalanced learning [7]. The degree to which one should balance the original data is hard to define or decide. In our proposed method, the imbalance level is defined, and the threshold is decided so as to generate the appropriate amount of augmented data. The assumptions that make imbalanced learning algorithms work better compared to learning from the original distribution are difficult to define clearly. Most standard algorithms assume balanced class distributions or equal misclassification costs. The combination of imbalanced data and small sample size presents new challenges. In this situation, all of the issues related to imbalanced learning are applicable. More importantly, high dimensionality hinders learning because of difficultly involved in forming conjunctions over the high degree of features with limited samples.

The rest of this article is organized as follows. Section 2 presents the details of the developed data augmentation method IMSDA. Section 3 shows the results of the proposed augmentation method applied to a real dataset from pulp and paper industry and the performance of failure detection which used supervised learning. Section 4 concludes the results and suggests some future directions.

## 2. The Proposed Data Augmentation Method for Multi-Sensor Stream Data

In this section, the proposed data augmentation method for imbalanced multi-sensor stream data is introduced and described, namely, IMSDA. Performing data augmentation without collecting new data is an effective and economic way to support a data-centric AI system. Before the novel method is presented, some basic background knowledge of our method is introduced.

### 2.1. The Basics of Method

The multi-sensor stream data are represented by multivariate time series matrix in the equations. In this section, a description of notations is given in Table 1. The complex numbers are not considered in this article.

#### 2.1.1. The Matrix Representation of Imbalanced Stream Data

R denotes the field of real numbers, respectively. The vector space of m×n matrix over R is denoted by $R^{m\times n}$. Multivariate time series can be denoted as $X=\left(x_{1}\left(t\right),x_{2}\left(t\right),\ldots x_{j}\left(t\right)\right)$, where t=1,2,…i. If $m_{s}$ data points for special time points are chosen from time series, it is a subset of X  which can be seen as a table of $m_{s}$ data points with dimension n. In other words, it can be represented as the form of $A_{s}$. $A_{s}$ denotes an $\;m_{s}\times n$ matrix. It can be represented by:

(1) $$A_{s}=\left(\begin{array}{cccc} x_{1}\left(1\right) & x_{1}\left(2\right) & \cdots & \;\;\;\;x_{m_{s}}\left(1\right)\\ x_{2}\left(1\right) & x_{2}\left(2\right) & \cdots & \;\;\;\;x_{m_{s}}\left(2\right)\\ \;\vdots & \;\vdots & \;\cdots & \vdots \\ x_{n}\left(1\right) & x_{n}\left(2\right) & \cdots & \;\;\;\;x_{m_{s}}\left(n\right) \end{array}\right)$$

#### 2.1.2. Singular Value Decomposition of Multivariate Stream Data Matrix

For $A\in \;R^{m\times n}$ , the singular value decomposition (SVD) of A is:

(2) $$A=U\Sigma V^{T}=u_{1}\sigma_{1}v_{1}^{T}+\ldots +u_{r}\sigma_{r}v_{r}^{T}$$

A is the real m×n matrix to decompose ; U is an m×m matrix; $u_{i}$ are left-singular vectors; sigma (Σ) is an m×n diagonal matrix; $V^{T}$ is the transpose of an n×n matrix $;\;v_{i}$ are right-singular vectors. The singular values in the diagonal matrix Σ are in decreasing order, $\sigma_{1}\geq \sigma_{2}\geq \ldots \geq \sigma_{r}$; $\sigma_{1}\;$ is the most important value [38]. SVD is a typical method of matrix decomposition which is computationally simple and accurate. All matrices have a SVD, which makes it more stable than other methods. As such, it is often used in a wide array of applications, including compressing, denoising, and data reduction. The imbalanced multi-sensor stream data can be seen as a matrix such as A, and it can be reconstructed from SVD.

#### 2.1.3. Cosine Similarity of Vectors

Cosine similarity is a measure of (dis)similarity between two non-zero vectors of an inner product space. The name derives from the term “direction cosine”: unit vectors are maximally “similar” if they are parallel and maximally “dissimilar” if they are orthogonal. It is defined as equal to the cosine of the angle between them, which is also the same as the inner product of the same vectors normalized to both have length 1. This is analogous to the cosine, which is unity (maximum value) when the segments subtend a zero angle and zero (uncorrelated) when the segments are perpendicular. The cosine similarity is particularly used in positive space, where the outcome is neatly bounded in [0,1]. Values close to 1 represent very similar vectors, while values close to 0 represent very different vectors.

The calculation of cosine similarity is:

(3) $$\cos=cos\left(a,b\right)=\frac{a\cdot \;b}{\left|\left|\;a\;\right|\right|\times \left|\left|\;b\;\right|\right|}$$

a· b is the dot product; $\left|\left|\;a\;\right|\right|$ is the norm of vector a; $\left|\left|\;b\;\right|\right|$ is the norm of vector b. The norm of a vector is calculated as:

(4) $$\left|\left|\;a\;\right|\right|=\sqrt{\underset{i}{\sum }a_{i}{}^{2}}$$

In practice, it is easier to calculate cosine of the angle between the vectors instead of the angle itself. The cosine similarity in this paper can be treated as a threshold which decides the amount and quality of generated data.

### 2.2. The IMSDA Method

IMSDA is developed for the imbalanced multi-sensor stream data augmentation. The description of the proposed method IMSDA is described as follows Algorithm 1.

**Algorithm 1:** The method of IMSDA**Input:** Raw imbalanced multi-sensor stream data labeled by failure (y = 1) or normal (y = 0). The variables (sensors) are $x_{1},x_{2},\ldots x_{n}$; n is the number of variables (sensors). **Output:** Augmented data for the minority class (y = 1). **Step 1:** Perform raw dataset preprocessing like normalization and delete some categorical variables which cannot be modeled. Store the processed data into a matrix, denoted as $A\in R^{m\times n}$, which has *m* samples and *n* variables. The variables can be represented by $x_{1},x_{2},\ldots x_{n}$. **Step 2:** The failure data and normal data can be treated as two classes. Calculate the degree of class imbalance.
(5)$$d=\frac{m_{s}}{m_{l}},\mathrm{where}d\in \left(0,1\right]$$where *m_s_* is the number of minority class (failure) samples, and *m_l_* is the number of majority class (normal) samples. Therefore, $m_{s}<m_{l}$$,\;m_{s}+m_{l}=m$. **Step 3:** If *d* < $d_{threshold}$, where
$d_{threshold}$ is the preset threshold for the maximum tolerated degree of class imbalance, then:      **a.** calculate the number of augmented data samples that needed to be generated from the minority class (failure class): (6)$$G_{d}=d_{threshold}\times m_{l}-m_{s}$$
The value of d*_threshold_* can be decided by domain experts. The amount of augmented data can also be calculated by: (7)$G_{\beta }=\left(m_{l}-m_{s}\right)\times \beta$
where *β* ∈ (0,1] is a parameter used to specify the desired balance level after data augmentation. *β* = 1 means that a fully balanced dataset is generated. The value of *β* can be decided by the models that we selected and domain expertise. *β* is different from ***d***, but both of them decide the amount of augmented data needed to be generated.      **b.** If
$m_{s}\geq n$, that means that the number of failure samples is bigger than the number of variables, then:
divide it into several smaller parts $m_{s_{1}},m_{s_{2}},\ldots ,\;m_{s_{i}}$, where $m_{s_{i}}<n$$,\;m_{s_{1}}+m_{s_{2}}+\ldots +m_{s_{i}}=m_{s}$.      If $m_{s}<n$ or $m_{s_{i}}<n$, that means that the number of failure samples is smaller than the number of variables, then: perform singular value decomposition of the multi-sensor stream data matrix. (8)$$A_{m_{s}}=U_{m_{s}}\Sigma_{m_{s}}V_{m_{s}}{}^{T}$$      Select $\left(1:k\right),k\in \left(1,\;m_{s}-1\right)$ of the matrix $V_{m_{s}}{}^{T}$; *k* is the size of singular values. The rows size of the augmented data is one row less than the original data.      **c.** Calculate cosine similarity matrix. The element of the matrix is calculated by:
(9)$$\cos=\frac{x_{r}\times a_{k}}{\left|\left|x_{r}\left|\left|\times \right|\right|a_{k}\right|\right|},\;\mathrm{where}\;cos\in \left(0,1\right]$$ where *x_r_* is any column of original data matrix; *a_k_* is any column in augmented multivariate data matrix. $\left|\left|\cdot \right|\right|$ represents the norm of a vector. The value of *cos* shows the approximation between the original column vector and the augmentation column vector.      **d.** Check the value of *cos*, and we can define a threshold *cos_threshold_* according to that. In practice, the value of cosine similarity is close to the correlation. We can also plot the data to check the difference between the curves of augmented data and original data intuitively. Thus, the size of *k* is decided. These data are treated as the augmented data.
**Step 4.** Calculate the imbalance degree: (10)$$d_{aug}=\;\frac{m_{s}+m_{aug}}{m_{l}}$$If $d_{aug}<d_{threshold}$, perform a loop in step 3. If $d_{aug}>d_{threshold}$, the loop ends. **Step 5:** Add the augmented data to the original data for supervised learning algorithm training.

For step 3, we have two choices to make a decision on the amount of augmented data to generate. The curves of the augmentation data which are created by the last k important singular values often appear more different from the curves of original data.

The key idea of IMSDA is to generate similar multivariate time series to approximate the raw data by using decomposition method and similarity discrimination. The level of imbalance and the amount of augmented data can be designed by domain experts depending on the situation. According to experience, the more balance the training data are, the better performance the supervised learning model can achieve. The minority class, which is positive labeled multivariate time series, is generated more by applying IMSDA. The augmented data not only increase the amount of training data but also still keep the property similar to the original abnormal time series data.

Due to the limited imbalanced multivariate time series data augmentation methods which can be used for this case, the comparison is between the performance of the supervised learning models trained by augmentation data and original data. The performance of the proposed method is described in the real case study.

## 3. Real Manufacturing Process Case Study

In order to test the effectiveness of the proposed method IMSDA for supervised failure detection in the paper manufacturing process, a real-world pulp and paper manufacturing case study was performed. As the real multi-sensor data from the mill is extremely imbalanced, the supervised failure detection is an imbalanced learning task. In this section, the procedures of how to apply our method and the results are described.

### 3.1. The Description of the Case

The paper machine is several meters long. It ingests raw materials at one end and produces reels of paper at the other end. The main raw material is wood, which is biological and, consequently, subject to natural variability. The chemical composition of wood chips depends on the type of the trees, the geographical location, the climate, and seasonal effects. The nonuniform characteristics of the wood chips propagate through the entire process and affect the final product quality. This is the long-term dependency, and this inherent variability makes the control of the papermaking processes a challenging task. During the processes, sometimes the paper breaks. If a sheet break happens, the entire process is often stopped, and the reel is taken out. The engineers need to check the machines to fix any problems, and the resumption can take a lot of time. The cost of the maintenance and the loss of production time is significant for the mills. The overall objective of the paper manufacturing process is to meet the specified paper quality and production targets while minimizing operation costs. From the operation perspective, the complex paper manufacturing process presents a variety of challenges and research topics [40,41].

**Figure 1:**
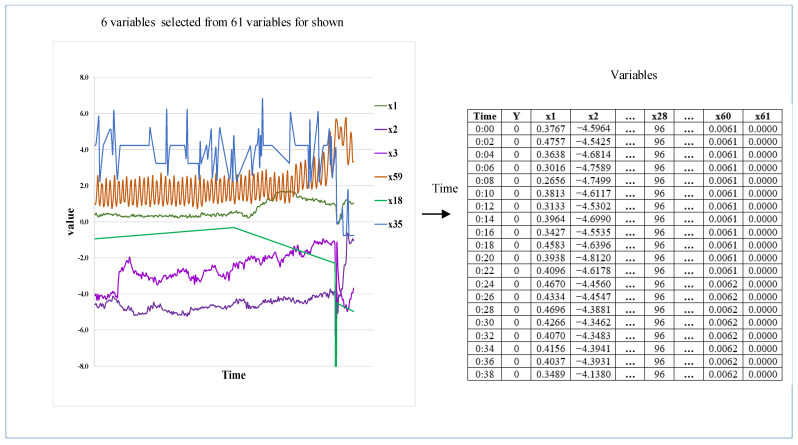
Original multi-sensor stream data from paper manufacturing processes.

The real paper manufacturing process case is from 2019 ProcessMinerTM Data Challenge organized by Informs QSR (Quality, Statistics, and Reliability Cluster of Institute for Operations Research and Management Sciences) [42]. The case is about the sheet break in a real paper and pulp mill, and the data are a multivariate time series collected by several sensors placed in different parts of the production line. The sensors measure raw materials, amount of pulp fiber, chemicals, blade type, couch vacuum, rotor speed, etc. The dataset is imbalanced, as the sheet break is a rare failure in the real paper making process, and an imbalanced multi-sensor stream dataset is taken from [29,36,43] which shows the specific name of the variables, while the challenge only used x1 to x61 to represent the variables. All the variables are continuous except x28 and x61. x61 is a binary variable, and x28 is a categorical variable that stands for the paper grade. The system’s status with regards to normal versus break is present in the SheetBreak column with the corresponding values as y = 0 and y = 1. The first column is time stamp. The observations are taken every 2 min, meaning that the rows in the data are 2 min apart with the timestamp present in the first column. Figure 1 shows the six real variables selected from the original multi-sensor stream data in the paper manufacturing process.

The basic statistical description of the raw dataset is shown in Table 2. The degree of class imbalance shows that the multivariate time series is extremely imbalanced (*d* is less than 1:100). There are limited failure samples but high dimensionality of the variables. The correlations of the variables are complex. Due to the above, building a supervised failure detection model with high precision and recall is quite challenging. There are six paper grades in the dataset. IMSDA is not applied to generate any data of paper grade 51, because there is only one failure in that data sample.

### 3.2. Results of Data Augmentation Method IMSDA

For simple and easy explanation, we only perform one loop of step 3 in IMSDA. In this way, we can know the different amount of augmented data created in one time. The augmented dataset and the degree of class imbalance are shown in Table 3. In the original paper grade 96 dataset, there are 72 rows of abnormal data. Because the number of rows is bigger than the number of columns, we should divide it into two datasets to make the number of rows less than the number of columns. The abnormal data are divided into 36 rows of data, and then the proposed method is applied. k is decided by the value of cos threshold. Different thresholds can lead to different k. In our case, we chose the threshold cos=0.5 for easy contrast among all of the paper grades. The degree of class imbalance of paper grade 96 is increased significantly.

**Figure 2:**
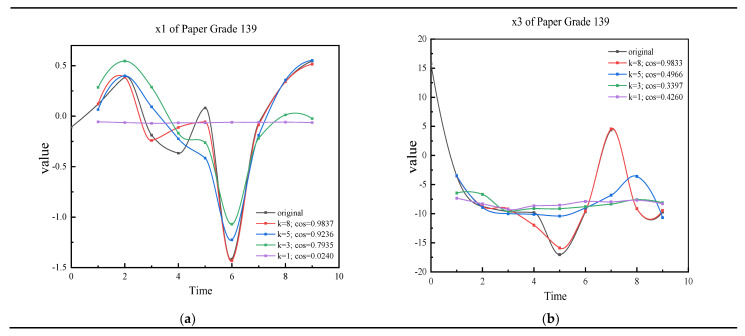
The curves of selected original data and the augmented data. (**a**) The curves of 139 original data and some of the augmented data (x1); (**b**) the curves of 139 original data and some of the augmented data (x3).

For domain experts, they can check all of the results qualitatively and quantitatively. Due to reasons of space, we only take the results of variable x1 and x3 in paper grade 139 as examples to show the similarity. The curves in Figure 2 qualitatively show the similarity of the augmented data and original data. The original abnormal time series of paper grade 139 and augmented time series with different k are plotted. In Figure 2a, it shows the augmented time series and original time series of variable x1. When k = 8, cos = 0.9837; the orange curve is almost the same as the original curve. When k = 1, the curve obviously shows the dissimilarity of the original time series and augmented time series. If k≥5, the augmented data can keep the temporal property of original x1. In Figure 2b, when k = 5, cos = 0.4966; we can see that the gray curve is a little different from the blue one. When k = 1, cos = 0.42; the yellow curve shows the big difference. Engineers can use this figure to help judge and decide the threshold. The curves in the figures intuitively show that IMSDA can keep the spatio-temporal property The results of other paper grades are similar to paper grade 139. The value of cosine similarity of the paper grades quantified shows the approximation of augmentation data. The cosine similarity also shows the correlation between the augmented time series and original time series. Although some variables can have higher thresholds, we need to make sure that all of the variables share the same threshold. Table 4 shows the value of cosine similarity of the paper grade 139 as an example. For each paper grade, there is a table, like Table 4, which saves the calculation results to refer to. In practice, the curves are used to check, and the threshold is mainly decided by the cosine similarity table quantitatively.

### 3.3. The Results of Supervised Failure Detection

Supervised learning algorithms are widely used for failure detection or binary classification problems. Logistic regression and random forest are efficient methods for classification and are extensively employed in industry. In this section, we present how we train these two models and evaluate the performance.

#### 3.3.1. The Performance Metrics in Imbalanced Learning

In imbalanced learning, the “Negative” is commonly referred to as the majority class, and the “Positive” is commonly referred to as the minority class. F1 score, precision, and recall are the commonly used in performance evaluation of models for imbalanced learning, while accuracy is the least important performance measurement. The related calculation formulas are listed in Table 5.

#### 3.3.2. The Supervised Failure Detection Models Trained by Augmentation Data

**Figure 3:**
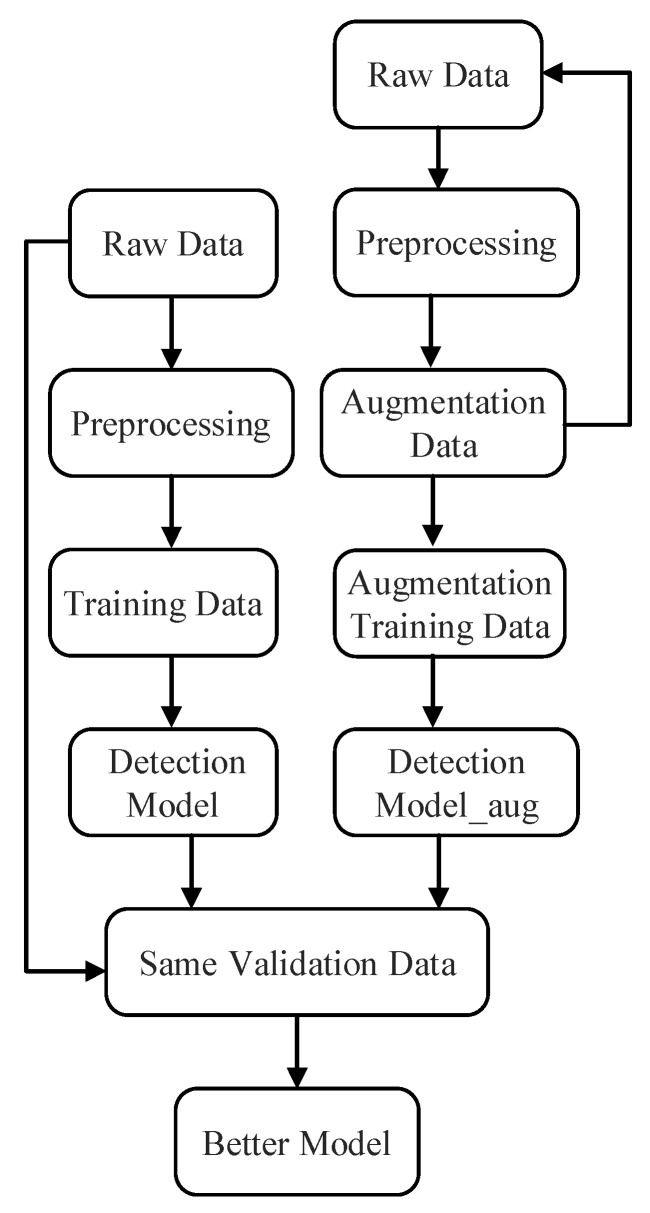
The flowchart for training and evaluating the supervised failure detection models.

The case is operated under Windows platforms, and the open-source library Anaconda is used. The data science-related packages are used to perform this task. The algorithms are trained with the augmented data and original data separately. Figure 3 shows the flowchart for training and evaluating the supervised failure detection models.

The augmentation data are added to the original data as training dataset. The preprocessing steps include standardization and removing the categorical variables are the same for both models. For comparison of the performance, both the model trained by the augmentation data and model trained by original data are validated by the same test dataset or validation dataset. The same validation dataset is real in practice and split from the original data. The better performance means the better supervised failure detection model.

(1) Logistic regression failure detection model trained by augmentation data

Logistic regression uses a loss function referred to as maximum likelihood estimation (MLE), which is a conditional probability. If the probability is greater than 0.5, the predictions will be classified as class 0. Otherwise, class 1 will be assigned. We choose the parameters random-state = 0, fold of cross-validation(cv) = 5, max_iter = 10,000. All of the other training parameters are the same for different paper grades. The algorithm is validated with the same validation dataset from the original data. All the main parameters are shown in Table 6. For easy comparison, the parameters stay the same for every paper grade except grade 139. For paper grade 139, if random-state = 0, there are no failures in the validation dataset. The random-state is changed to 1, while other parameters stay the same as other paper grades.

The dataset is split into training set, test set, and validation set. The training set is used to fit the model. The test set is used to provide an unbiased evaluation of the final model fit on the training dataset. The validation set is used to provide the final results. The final model can be fit on the aggregate of the training and validation datasets. The augmentation data are only used for training the models; they are not used for testing the performance of detection model. That means all of the clf and clf_aug models are tested strictly by the same validation set split from the raw dataset. The performance of the logistic regression models trained by the original data or augmentation data is shown in Table 7.

The results show that the logistic regression model trained by augmentation data performs better than the model trained without augmentation. For paper grade 96 and 82, the models trained by augmentation data perform much better than the model trained by raw data. There are 5 true failures in the paper grade 118 validation dataset. The clf118_aug predicted 6 failures, and clf118 predicted 0 failures. All of the measurements of the performance are improved, including the accuracy. For paper grade 139, there are two failure data samples in the validation dataset. The model clf139 failed to detect any abnormality, while the model clf139_aug trained by augmentation data detect both of them. That is why all of the performance is equal to 1. For paper grade 112, there is only one row of failure data. The model trained by the original data failed to detect the abnormal behavior, but the model trained by the augmentation data can detect the failure. That is why precision = 0, recall = 0 for the original data. Even the model clf112 did not detect anything, the accuracy is 0.996. However, the accuracy is not an important performance evaluation in the extreme imbalanced learning problem.

(2) Random forest failure detection model trained by augmented data

Random forest or random decision forest is an ensemble method for classification, regression, and other tasks. For classification tasks, the output of random forest is the class selected by most trees. The parameters of the training process are shown in Table 8. The original data are split into train dataset and test dataset. The test dataset is used to evaluate the performance of both two models.

The results in Table 9 show that the random forest model trained by augmentation data performs better for most paper grades. For paper grade 96, although the value of accuracy and recall are decreased, the F1-score = 1, which means that the augmentation model detects all of the failures but generates more false alarms. However, the F1_score is the most important value. Augmentation models can improve the value of recall for all paper grades.

The results of both logistic regression and random forest show data augmentation can improve the generalization of supervised failure detection models. It is critical to make the complex manufacturing process more efficient and reduce loss.

## 4. Conclusions and Discussion

In this section, our research work is concluded, and the advantages and disadvantages of our method are discussed. Some future research directions of our work are also given.

In the view of data-centric AI, data quality is crucial for the success of AI systems. Data augmentation is a key technique for effective machine learning models and for improving their generalization in practice. In our study, the research challenges combine multi-sensor stream data augmentation and imbalanced learning. Multi-sensor stream data augmentation is problem-dependent, and there is no best way of performing multivariate time series data augmentation. The proposed method, IMSDA, is proposed to generate good quality of synthetic failure data to balance the original dataset. In the real case study, the proposed method is employed. The real dataset is about all the signals collected by the sensors along the production line in complex paper manufacturing processes. It is imbalanced because of the lack of failure data, and it is high dimensional due to the various variables. Logistic regression and random forest, which are supervised learning models, are trained by the original data and the augmented data. The results show that the models trained by augmentation data perform better than the models trained by the original data. The results also show the proposed method can generate high-quality augmented multivariate time series data. This data augmentation strategy significantly improves the supervised model generalization on failure detection in complex manufacturing processes. It shows the potential of data augmentation to improve the performance of anomaly detection by supervised machine learning.

In the data augmentation process, the same threshold is decided for each paper grade for easy comparison. However, the threshold can be decided depending on the situation in practice. The supervised learning can be run for each value of cos to check which can help the model to perform better. The balance between cost of iterations and economic model should be kept based on practical experience or domain knowledge in practice. The calculation of our method is incredibly fast, and the model can help to perform real-time update of the algorithm. If more real abnormal data in real-world applications could be collected, our models could be frequently updated. In the future, it is worthwhile to spend more time in carrying out iterations to find the optimal threshold or parameters. More experiments should be organized to validate the effects of the proposed data augmentation method on both supervised learning and semi-supervised learning. The data-centric approach may play the same role as the model-centric approach. Some steps of the proposed method are decided by domain expertise, and more research work should be focused on how to make it automatic like the various models.

## Figures and Tables

**Table 1:** The description of the notations.

Notation	Description
R	The field of real numbers
X	Multivariate time series
A	Matrix (multivariate time series for special time)
U	Matrix
Σ	Diagonal matrix
V	Matrix
u	Left-singular vectors
σ	Singular value
v	Right-singular vectors
cos	Cosine similarity (threshold)
d	The degree of class imbalance
β	The desired balance level
G	The size of augmented data
m	The rows of the matrix
n	The columns of the matrix (the number of variables)

**Table 2:** The statistic description of original multi-sensor stream data.

Paper Grade	Normal	Failure	The Degree of Class Imbalance
96 *	6502	72	0.0111
82	4360	18	0.0041
118	2631	15	0.0057
139	1797	10	0.0056
112	1230	5	0.0041

* Means that rows of failure data are more than columns of dataset.

**Table 3:** The description of dataset after data augmentation.

Paper Grade	cos	k	Normal	Abnormal	The Degree of Class Imbalance
96 *	0.5	18–35	6502	1332	0.2049
82	0.5	14–17	4360	86	0.0197
118	0.5	10–14	2631	85	0.0323
139	0.5	6–9	1797	46	0.0256
112	0.5	4	1230	9	0.0073

* Means that rows of failure data are more than columns of dataset.

**Table 4:** The value of cosine similarity of the paper grade 139.

	*k* = 9	*k* = 8	*k* = 7	*k* = 6	*k* = 5	*k* = 4	*k* = 3	*k* = 2	*k* = 1
**x1**	1.0000	0.9837	0.9522	0.9521	0.9236	0.9020	0.7935	0.6554	**0.0240**
**x2**	1.0000	0.9979	0.9915	0.5518	**0.3680**	0.3487	0.3567	0.2287	0.2072
**x3**	1.0000	0.9883	0.8937	0.5886	**0.4966**	0.3556	0.3397	0.4897	0.4260
**⁝**	⁝	⁝	⁝	⁝	⁝	⁝	⁝	⁝	⁝
**x60**	1.0000	0.9860	0.9800	0.9114	0.8948	0.8915	0.5730	0.3125	0.5484

**Table 5:** Performance metrics for imbalanced learning.

Notation	Description
Accuracy	(TP + TN)/(TP + FP + TN + FN)
Recall	TP/(TP + FN)
Precision	TP/(TP + FP)
F1 score	2 × Precision × Recall/(Precision + Recall)

TP: True Positive; TN: True Negative; FP: False Positive; FN: False Negative.

**Table 6:** The parameters of training model.

Rank	Parameters	Values
1	cv(cross-validation)	5
2	* Random-state	0
3	Max_iter	10,000
4	Train dataset	0.6
5	Test dataset	0.2
6	Validation dataset	0.2

* For paper grade 139, random-state = 1.

**Table 7:** The performance of logistic regression.

Paper Grade	Model	Accuracy	F1 Score	Precision	Recall
82	Original data trained model clf82	0.997	0.666	0.558	0.666
	Augmentation data trained model clf82_aug	0.998	0.857	0.638	1
96	Original data trained model clf96	0.993	0.400	0.340	0.330
	Augmentation data trained model clf96_aug	0.995	0.530	0.542	0.444
112	Original data trained model clf112	0.996	0.004	0	0
	Augmentation data trained clf112_aug	1	1	1	1
118	Original data trained model clf118	0.991	0	0.035	0
	Augmentation data trained model clf118_aug	0.998	0.909	0.998	1
139	Original data trained model clf139	0.994	0	0.02	0
	Augmentation data trained model clf139_aug	1	1	1	1

**Table 8:** The parameters of training model.

Rank	Parameters	Values
1	Random-state	0
2	Train dataset	0.8
3	Test dataset	0.2

**Table 9:** The performance of random forest.

Paper Grade	Model	Accuracy	F1 Score	Precision	Recall
82	Original data trained model rf82	0.997	0.571	0.667	0.500
	Augmentation data trained model rf82_aug	0.995	1.000	0.500	1.000
96	Original data trained model rf96	0.995	0.769	0.833	0.714
	Augmentation data trained model rf96_aug	0.101	1.000	0.012	1.000
112	Original data trained model rf112	0.996	1.000	0.000	0.000
	Augmentation data trained model rf112_aug	0.996	1.000	0.000	0.000
118	Original data trained model rf118	0.994	0.400	1.000	0.250
	Augmentation data trained model rf118_aug	0.998	1.000	1.000	0.750
139	Original data trained model crf139	1.000	1.000	1.000	1.000
	Augmentation data trained rf139_aug	0.980	1.000	0.430	1.000

## Data Availability

The link of the dataset is in the book “Understanding Deep Learning: Application in Rare Event Prediction” written by Chitta Ranjan and published by Connaissance in 2020.
